# Status of ambulatory blood pressure monitoring and home blood pressure monitoring for the diagnosis and management of hypertension in the US: an up-to-date review

**DOI:** 10.1038/s41440-022-01137-2

**Published:** 2023-01-05

**Authors:** Maria Cepeda, Patrick Pham, Daichi Shimbo

**Affiliations:** Department of Medicine, Columbia University Irving Medical Center, New York, NY USA

**Keywords:** Hypertension, Blood pressure measurement, Cardiovascular disease, Ambulatory blood pressure monitoring, Home blood pressure monitoring

## Abstract

The diagnosis and management of hypertension has been based on the measurement of blood pressure (BP) in the office setting. However, data have demonstrated that BP may substantially differ when measured in the office than when measured outside the office setting. Higher out-of-office BP is associated with increased cardiovascular risk independent of office BP. Ambulatory BP monitoring (ABPM) and home BP monitoring (HBPM) are validated approaches for out-of-office BP measurement. In the 2015 and 2021 United States Preventive Services Task Force (USPSTF) reports on screening for hypertension, ABPM was recommended as the reference standard for out-of-office BP monitoring and for confirming an initial diagnosis of hypertension. This recommendation was based on data from more published studies of ABPM vs. HBPM on the predictive value of out-of-office BP independent of office BP. Therefore, HBPM was recommended as an alternative approach when ABPM was not available or well tolerated. The 2017 American College of Cardiology (ACC)/American Heart Association (AHA) BP guideline recommended ABPM as the preferred initial approach for detecting white-coat hypertension and masked hypertension among adults not taking antihypertensive medication. In contrast, HBPM was recommended as the preferred initial approach for detecting the white-coat effect and masked uncontrolled hypertension among adults taking antihypertensive medication. The current review provides an overview of ABPM and HBPM in the US, including best practices, BP thresholds that should be used for the diagnosis and treatment of hypertension, barriers to widespread use of such monitoring, US guideline recommendations for ABPM and HBPM, and data supporting HBPM over ABPM.

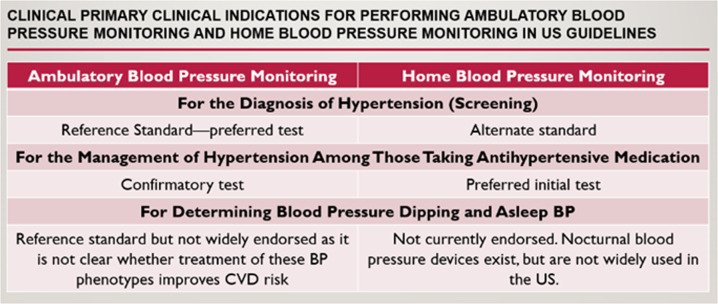

## Introduction

Traditionally, hypertension has been primarily determined in the office setting by the measurement of blood pressure (BP) [1]. United States (US) guidelines and scientific statements recommend measuring BP in the clinical office setting to identify adults with hypertension and to evaluate BP control among individuals with hypertension, including those taking antihypertensive medication [1–3]. This recommendation is supported by substantial data, demonstrating that higher office BP is associated with CVD and target end-organ damage, and BP goals in randomized trials have been based on office BP [1]. In the past, office BP has been recommended to be determined by using the auscultatory method with a mercury sphygmomanometer, but this has largely been replaced with recommendations to use an oscillometric device [1].

Many individuals have a BP that is different outside versus inside the office [4, 5]. Compared with office BP, out-of-office BP has been demonstrated to be more strongly associated with target end-organ damage and cardiovascular disease events [6–9]. Most guidelines recommend conducting out-of-office BP monitoring to confirm the presence or absence of high office BP for the diagnosis and management of hypertension [10–12]. It is estimated that 46% of US adults (or approximately 104 million) meet the 2017 American College of Cardiology (ACC)/American Heart Association (AHA) BP guideline criteria for out-of-office BP monitoring [13]. There are two main methods for out-of-office BP monitoring: ambulatory BP monitoring (ABPM) and home BP monitoring (HBPM) [5]. ABPM, which has existed for more than 50 years, is a fully automated method where BP is measured over a 24-hour period. HBPM is a newer approach and involves the self-measurement of BP by an individual outside of the office at his or her home, typically over several days to a week.

In the current review, we provide a clinical perspective for using ABPM and HBPM for the diagnosis and management of hypertension among US adults.

## Overview of ABPM

ABPM is a type of out-of-office BP monitoring, typically assessed using the oscillometric method, that occurs outside of the office setting. ABPM is usually performed over a 24-hour period, although longer monitoring periods have been performed [1, 5]. ABPM provides a larger number of readings than office BP measurements and allows for the assessment of BP outside of the office setting over a 24-hour period. Studies have demonstrated that higher BP on ABPM is a stronger predictor of target end-organ damage and CVD events than office BP [8]. In clinical practice, ABPM is used to determine the presence of white-coat hypertension (high office BP without high out-of-office BP among those not taking antihypertensive medication) and masked hypertension (high out-of-office BP without high office BP among those not taking antihypertensive medication), white-coat effect (high office BP without high out-of-office BP among those taking antihypertensive medication), masked uncontrolled hypertension (high out-of-office BP without high office BP among those taking antihypertensive medication), nocturnal hypertension (high nighttime BP), and BP dipping patterns (i.e., dipping, nondipping, extreme dipping, and reverse dipping) [1]. ABPM is used to monitor antihypertensive medication treatment efficacy [5], and can also evaluate postural, postprandial, and drug-induced hypotension as well as hypotension from autonomic dysfunction [1, 5].

## Best practices for ABPM

### Device selection

Only ABPM devices that have been validated for accuracy should be used. There are several US websites that provide lists of BP measurement devices that have been previously validated. The BP Validated Device Listing (VDL™) website lists validated BP devices that can be used in clinical practice in the US [14]. An independent review committee composed of hypertension experts decides whether a BP device has satisfied the criteria for inclusion on the VDL. For a device to be on the VDL, validation data must be provided, preferably generated externally and published in peer-review format [15]. The following are acceptable for supportive data: a peer-reviewed publication, independent third-party validation testing by a qualified entity, or validation testing conducted by the manufacturer with full documentation of the methodology employed and how the data were produced [15].

### Cuff selection

The patient’s arm circumference should be measured on the mid-upper arm, between the acromion and olecranon. Appropriate-sized BP cuffs should be used for ABPM. BP cuffs should be placed on the bare skin of the nondominant arm, and the patient’s BP cuff should be at a resting level with their right atrium.

### Monitoring period and frequency of BP measurements

Typically, the ABPM device is programmed to obtain measurements every 15 to 30 min throughout a 24-hour period. ABPM measurements may be taken less frequently (i.e., hourly) during the asleep period to decrease sleep disturbance [1].

## BP thresholds on ABPM

In the past, thresholds in the US for having high BP on ABPM were defined as the following: ≥135/85 mm Hg for daytime BP, ≥ 130/80 mm Hg for 24-hour BP and ≥ 120/70 mm Hg for nighttime BP [5]. These ABPM thresholds correspond to an office BP ≥ 140/90 mm Hg and are consistent with the ABPM thresholds recommended in international guidelines [11, 12].

In the 2017 ACC/AHA BP guidelines, the office BP threshold for hypertension status was lowered to ≥130/80 mm Hg [10]. Consequently, thresholds for having high BP on ABPM, which correspond to an office BP ≥ 130/80 mm Hg, were defined in the US as follows: ≥130/80 mm Hg for daytime BP, ≥ 125/75 mm Hg for 24-hour BP, and ≥110/65 mm Hg for nighttime BP [10, 16]. Although the 2017 ACC/AHA BP guideline recommends conducting ABPM over an entire 24-hour period, it also recommends using daytime BP rather than 24-hour BP or nighttime BP for the diagnosis and management of hypertension [10]. Therefore, this recommendation makes it unclear whether ABPM should be performed only during the daytime period versus over an entire 24-hour period.

### Potential limitations of ABPM

Despite US guidelines supporting ABPM, ABPM is not widely available in the US and is not commonly used in clinical practice [5, 17]. We previously reported that clinician-level barriers to ABPM in the US include the need for staff training, time constraints in patient preparation, and poor access to equipment and specialists to whom providers could refer their patients for ABPM [18]. ABPM devices usually cost over $2,000 USD each, and when ABPM is reimbursed, the amount is low [17, 19]. There are also patient-level barriers. Low patient tolerability to ABPM has been reported [20, 21]. One study found that side effects associated with ABPM ranged from bruising (7%) to the device awakening the person during sleep (70%) [20]. In a more recent study that we conducted with investigators from the Centers for Disease Control and Prevention (CDC), 55% of participants reported that ABPM interfered with their sleep [21].

## Overview of HBPM

HBPM is another type of out-of-office BP monitoring that consists of individuals self-measuring their BP at home typically using an oscillometric device [1, 5]. Several studies have shown that BP on HBPM has a stronger association with CVD events than office BP [6, 7, 22, 23]. The 2017 ACC/AHA BP guideline stated that it is reasonable to use HBPM to identify white-coat hypertension and masked hypertension among individuals not taking antihypertensive medication and that it may be reasonable to use HBPM to identify white-coat effect and masked uncontrolled hypertension among individuals taking antihypertensive medication [10]. It is also reasonable to use HBPM to monitor the progression of white-coat hypertension to sustained hypertension [10]. Other indications for HBPM include determination of BP control during hypertension treatment, exclusion of false resistant hypertension (having resistant hypertension based on office BP but with controlled out-of-office BP), and use of HBPM as an approach to empowering patients in BP management, including improving antihypertensive medication adherence [10, 24]. In the US, HBPM is more widely available and less expensive to conduct than ABPM. Using nationally representative data from the National Health and Nutrition Examination Survey (NHANES), 2009–2010, Ostchega et al. estimated that 21.7% of US adults reported using HBPM in the past year, and 14.5% reported engaging in monthly or more frequent HBPM [25]. Among adults with hypertension, 36.6% reported engaging in monthly or more frequent HBPM [25]. Evidence also indicates that HBPM is better tolerated by patients than ABPM [23, 24]. Furthermore, specialists are not needed to implement HBPM, and it can be easily implemented in primary care practices in the US. Therefore, HBPM is a more feasible approach than ABPM for diagnosing and managing hypertension.

## Best practices for HBPM

### Device selection

Similar to ABPM device selection, it is important to use validated HBPM devices. There are many HBPM devices widely available to patients either in pharmacies or online in the US. Evidence indicates that many devices sold online have not been properly validated [26]. Similar to ABPM devices, a list of validated HBPM devices sold in the US can be found on the BP Validated Device Listing (VDL™) website [14]. HBPM devices can either be automatic (i.e., can take multiple readings with a single activation) or semiautomatic (i.e., can take only 1 reading with each activation). It is recommended that appropriate-sized brachial cuffs are used for HBPM. Using data from the National Health and Nutrition Examination Survey (NHANES), 2015–2020, Jackson et al., 51% of US adults overall, including 65% of those aged 18–34 years and 84% of those with obesity, needed large or extra-large cuffs [27]. Given that large or extra-large cuffs do not come routinely with many HBPM devices, it is likely that in routine clinical practice, BP is being measured inaccurately among adults with large or extra-large sized arms who are using HBPM devices. It is critical that patients and health care providers in the US be educated about appropriate cuff selection and whether HBPM devices come with large and extra-large sized cuffs.

Wrist devices with a cuff are not recommended for HBPM, as only a few have been validated [26]. Wrist devices with a cuff may be useful for individuals whose arms do not fit into available brachial cuff sizes [1, 24]. HBPM devices that store readings avoid issues related to the person incorrectly documenting the measurements [1]. BP readings should be printed or transferred electronically to health care providers [24]. BP devices that can also transmit data wirelessly to smartphone applications are now widely available [1, 24]. During the COVID-19 pandemic, the use of home BP telemonitoring expanded in the US as in-person office visits decreased. A challenging approach is the transfer of BP data from these applications directly into the electronic health record, making the data more accessible to the patient’s health care team so that the management of BP control is more efficient [24]. The use of nocturnal HBPM devices that measure BP during sleep has recently been utilized, and data suggest that these HBPM devices may provide similar mean asleep BP and nondipping BP status to those obtained by ABPM [28, 29]. However, nocturnal HBPM devices are not commonly used in the US either in research or in clinical practice. Currently, no US hypertension guideline recommends using nocturnal HBPM devices to determine nighttime BP or BP dipping phenotypes.

### Monitoring period and frequency of BP measurements

Patients and providers should be instructed and educated on the use of HBPM. Patient-centric and provider-centric training materials can be found on the US Target: BP Initiative and Centers for Disease Control and Prevention/Centers for Medicare & Medicaid Services Millions Heart Initiative’s websites [30, 31]. Proper HBPM techniques include the following: (1) the patient’s arm should be supported, e.g., resting on a desk, (2) the cuff should be placed directly above the antecubital fossa, and (3) the center of the bladder should be placed over the artery of the upper arm. The preferred HBPM period is 7 days, with 2 AM and 2 PM readings performed each day [24]. A minimum period of 3 days with 2 AM and 2 PM readings is also sufficient [32–34]. Once BP is controlled, 1 to 3+ days of readings is reasonable [35]. For each monitoring period, the average of all home BP readings should be calculated [24].

## BP thresholds on HBPM in the US

In the past, thresholds for having high BP on HBPM were defined as the following: ≥135/85 mm Hg, which corresponded to an office BP ≥ 140/90 mm Hg [5]. With the 2017 ACC/AHA BP guideline, the BP thresholds on HBPM were newly defined as ≥130/80 mm Hg, which corresponds to an office BP ≥ 130/80 mm Hg [10, 16].

## Importance of HBPM cointerventions

The use of HBPM is associated with a reduction in BP and improved BP control, and the benefits of BP lowering with HBPM are greatest when it is conducted with HBPM cointerventions, which include education, behavioral change management, communication of treatment recommendations to patients, telemonitoring and telecounseling, nurse or pharmacist management of antihypertensive medication, and/or prescription monitoring [24, 36]. Compared to usual care, the use of HBPM alone leads to reductions in systolic BP and diastolic BP at 6 months but no reductions in systolic BP and diastolic BP at 12 months. However, there are reductions in systolic BP and diastolic BP and improved BP control at 12 months when HBPM use is accompanied by HBPM cointerventions [24, 36]. These data highlight the importance of delivering cointerventions when implementing HBPM in clinical practice.

## Potential limitations of HBPM

There are several challenges associated with HBPM in the US. Patients and providers are not well informed about which HBPM devices are validated [18, 24]. Some HBPM devices do not automatically record BP measurements. Therefore, there is an increased reliance on patients to document their own readings, and patients may not report their BP accurately [37]. Additionally, HBPM may lead to the patient’s preoccupation with their BP, which may lead to anxiety [24]. Another challenge is that long-term adherence to HBPM over months and years could pose a problem, and therefore, ongoing educational training and support are necessary. In the US, effective January 1, 2020, 2 Current Procedural Terminology (CPT) reimbursement codes have been added to support the performance of HBPM among patients: CPT 99473, self-measured BP with a device validated for clinical accuracy, along with patient education/training and device calibration; and CPT 99474, separate self-measurements of 2 readings, 1 min apart, twice daily over a 30-day period (minimum of 12 readings), collection of data reported by the patient or caregiver to the physician or other qualified health care professional, with the report of average systolic BP and diastolic BP and subsequent communication of a treatment plan to the patient. However, the costs for purchasing devices are typically not reimbursed by insurance companies. Therefore, HBPM may be inaccessible to individuals with a low income. Finally, in the US, patients with hypertension may not have access to HBPM cointerventions.

## ABPM or HBPM: which approach is preferred for hypertension diagnosis and management in the US?

US guidelines, including the 2015 United States Preventive Services Task Force (USPSTF) report, have endorsed ABPM as the reference standard for out-of-office BP monitoring, as more studies have examined associations of out-of-office BP with target end-organ damage and CVD using ABPM than using HBPM [3, 10]. HBPM has been consistently recommended as an alternative approach, which can be performed if ABPM is not available or poorly tolerated by the patient [24]. More recently, a 2020 systematic review, conducted for an update to the 2015 USPSTF report on hypertension screening, stated that it “solely accepted ABPM as the gold standard for hypertension diagnosis” due to the smaller evidence base supporting HBPM [38–41]. In 2021, USPSTF reaffirmed their endorsement of ABPM as the reference standard for out-of-office BP monitoring [2]. The 2015 and 2021 USPSTF reports focused on excluding white-coat hypertension among adults with high office BP and did not make any recommendations on using out-of-office BP monitoring to exclude masked hypertension among adults without high office BP [2, 3].

Table 1 shows the 2017 ACC/AHA BP guideline recommendations on ABPM and HBPM, which focused on detecting white-coat hypertension and masked hypertension among individuals not taking antihypertensive medication and white-coat effect and masked uncontrolled hypertension among individuals taking antihypertensive medication [10]. For detecting white-coat hypertension and masked hypertension, ABPM was recommended as the preferred initial approach. In contrast, HBPM was recommended as the preferred initial approach for detecting the white-coat effect and masked uncontrolled hypertension. Although the 2017 ACC/AHA BP guideline [10] endorsed ABPM as the “preferred” option and that HBPM was a “less desirable alternative,” the rationale for recommending HBPM over ABPM for adults taking antihypertensive medication was that HBPM is a more practical approach than ABPM for repeat assessments over time. However, for adults taking antihypertensive medication, the 2017 ACC/AHA BP guideline also recommended that ABPM be used to confirm the results on HBPM, suggesting that HBPM alone may be insufficient for individuals taking antihypertensive medication [10].

**Table 1:** Recommendations for conducting ABPM and HBPM in the 2017 American College of Cardiology/American Heart Association BP Guideline

Out-of-office BP measurements are recommended to confirm the diagnosis of hypertension and for the titration of antihypertensive medication in conjunction with telehealth counseling or clinical interventions. **COR I and LOE A**^**SR**^
In adults with an untreated systolic BP > 130 mmHg but <160 mm Hg or diastolic BP > 80 mm Hg but <100 mm Hg, it is reasonable to screen for the presence of white-coat hypertension by using either daytime ABPM or HBPM before the diagnosis of hypertension is made. **COR IIa and LOE B-NR**
In adults with white-coat hypertension, periodic monitoring with either ABPM or HBPM is reasonable to detect the transition to sustained hypertension. **COR IIa and LOE C-LD**
In adults being treated for hypertension with office BP readings, not at goal and HBPM readings suggestive of a significant white coat effect, confirmation by ABPM can be useful. **COR IIa and LOE C-LD**
In adults with untreated office, BPs that are consistently between 120 mm Hg and 129 mm Hg systolic or between 75 mm Hg and 79 mm Hg for DBP, screening for masked hypertension with HBPM (or ABPM) is reasonable. **COR IIa and LOE B–NR**
In adults on multiple-drug therapies for hypertension and office BPs within 10 mm Hg above goal, it may be reasonable to screen for white coat effect with HBPM (or ABPM). **COR IIb and LOE C-LD**
It may be reasonable to screen for masked uncontrolled hypertension with HBPM in adults being treated for hypertension and office readings at goal, in the presence of target organ damage or increased overall CVD risk. **COR IIb and LOE C-EO**
In adults being treated for hypertension with elevated HBPM reading suggestive of masked uncontrolled hypertension, confirmation of the diagnosis by ABPM might be reasonable before intensification of antihypertensive drug treatment. **COR IIb and LOE C-EO**

*ABPM* ambulatory blood pressure monitoring, *BP* bloodpressure, *COR* class of recommendation, *CVD* cardiovascular disease, *EO* expertopinion, *HBPM* home blood pressure monitoring, *LD* limited data, *LOE* level ofevidence, *NR* non-randomized data, *SR* systematic review, *COR 1* is recommended, *COR IIa* is reasonable, *COR IIb* may be considered, *COR III* no benefit or possible harm, *LOE A* high quality evidence, *LOE B* moderate quality evidence, *LOE C-LD* limited data, *LOE C-EO* expert opinion.

One unique recommendation from the 2017 ACC/AHA BP guideline, which was not consistent with the prior US and international guidelines, is that ABPM should be performed to exclude white-coat hypertension and masked hypertension among adults with specific office BP criteria (Table 1) but only after a 3-month trial of lifestyle modification. Our prior work has shown that few participants who meet office BP criteria for the screening of white-coat hypertension and masked hypertension had ideal lifestyle factors [42]. Using data from 2 US studies, the Coronary Artery Risk Development in Young Adults (CARDIA) study and the Jackson Heart Study (JHS), we found that 15.5% of CARDIA participants and 3.6% of JHS participants had 3 or more ideal lifestyle factors among participants who met office BP criteria for white-coat hypertension screening, and 22.6% of CARDIA participants and 4.7% of JHS participants had 3 or more ideal lifestyle factors among participants who met office BP criteria for masked hypertension screening. Therefore, most adults will need to be recommended for a 3-month trial of lifestyle modification prior to ABPM, and there may be a delay in the exclusion of white-coat hypertension and masked hypertension for individuals who do not have ideal lifestyle factors.

## What is the evidence indicating that ABPM is superior to HBPM?

Although some studies have shown that ABPM may be prognostically superior to HBPM [43], the data are too scarce overall to firmly conclude whether ABPM or HBPM is superior for assessing CVD risk. In our prior systematic review of 9 cohort studies, there was insufficient evidence indicating whether ABPM is superior to HBPM or vice versa for the association of BP with CVD [44]. Therefore, there is uncertainty as to whether ABPM or HBPM should be the reference standard for out-of-office BP monitoring. There are important scientific implications of ABPM being the de facto reference standard. As ABPM has been considered the reference standard for out-of-office BP monitoring, many studies have assumed that ABPM has perfect accuracy (i.e., 100% sensitivity and 100% specificity) when assessing the diagnostic accuracy of HBPM [38, 45, 46]. These studies have concluded that HBPM is insufficient for detecting high BP measured by ABPM. However, if HBPM was assumed to have perfect accuracy, ABPM would be insufficient for detecting high BP measured by HBPM since many individuals who have high BP on HBPM do not have high BP on ABPM, and many who do not have high BP on HBPM have high BP on ABPM. Studies have also compared the cost-effectiveness of HBPM versus ABPM for diagnosing hypertension [47, 48]. For example, Lovibond et al. estimated cost-effectiveness in a hypothetical UK primary-care population ≥40 years of age with high office BP. ABPM, which was assumed to have perfect accuracy, was the most cost-effective strategy for the diagnosis of hypertension [48]. However, when the sensitivity and specificity of HBPM were assumed to be equal to those of ABPM, HBPM was the most cost-effective strategy.

**Fig. 1:**
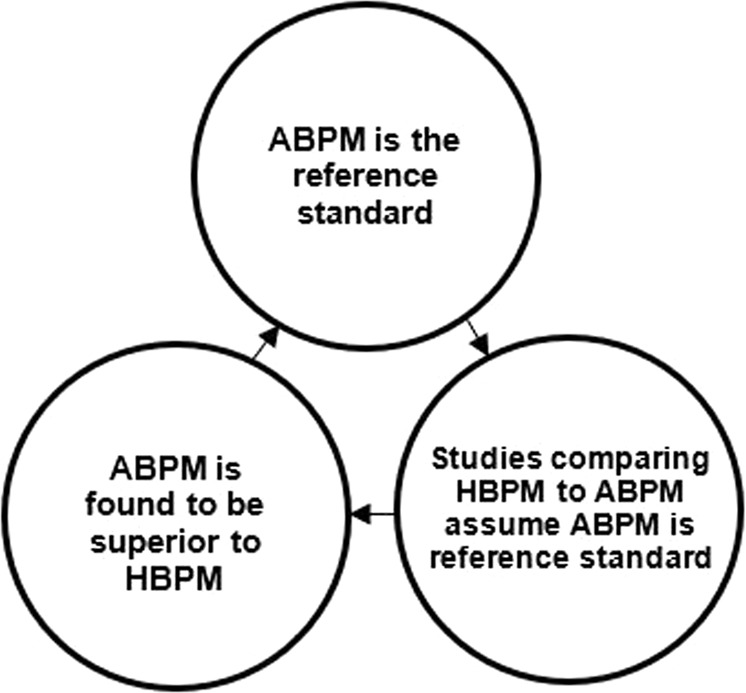
Circular reasoning assuming ABPM to be a reference standard

Figure 1 shows the circular reasoning that occurs when ABPM is assumed to be the reference standard for out-of-office BP monitoring.

**Fig. 2:**
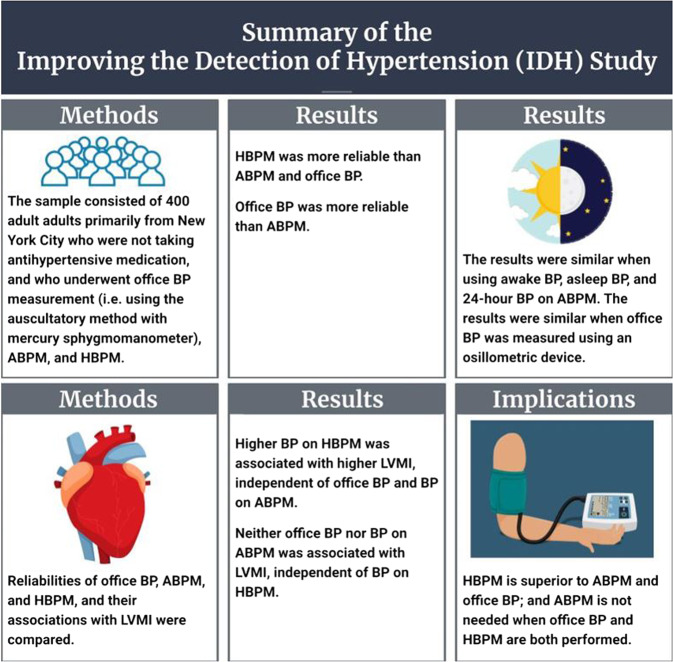
Summary of the Improving the Detection of Hypertension (IDH) Study. ABPM ambulatory blood pressure monitoring. BP blood pressure. HBPM home blood pressure monitoring. LVMI left ventricular mass index

Our recent study compared office BP, BP measured by ABPM, and BP measured by HBPM when all values were assessed using guideline-recommended approaches [49]. In the Improving the Detection of Hypertension (IDH) study, funded by our prior Program Project Grant (PPG) from the National Heart, Lung, and Blood Institute at the National Institutes of Health, we compared the reliability of office BP (3 visits with 3 readings/visit), BP measured by ABPM (every 30 minutes over a 24-hour period), and BP measured by HBPM (2 AM and 2 PM readings per day over 7 days), and the associations of each BP measure with the left ventricular mass index (LVMI), a validated measure of target end-organ damage. The IDH study consisted of 400 adult community-dwelling adults primarily from Upper Manhattan (mean age 41 years; 60% female; 15% non-Hispanic white, 14% non-Hispanic Black, 64% Hispanic, 6% Asian, and 1% other) who were not taking antihypertensive medication. Office BP was measured using the auscultatory method with a mercury sphygmomanometer during 3 visits with 3 readings/visit. ABPM was performed using a Spacelabs 90207 oscillometric device every 30 minutes over two 24-hour periods. HBPM was performed using an Omron HEM-790IT or HEM-791IT oscillometric device with 2 AM and 2 PM readings per day over three 7-day periods. The reliabilities (i.e., intraclass correlation coefficients, ICCs) of HBPM, office BP, and awake BP on ABPM were 0.938, 0.894, and 0.834, respectively, for systolic BP. In separate models that adjusted for age, sex, Black race, Hispanic ethnicity, body mass index (BMI), and diabetes status, the estimated difference in LVMI per 10 mm Hg higher systolic BP was 2.52 g/m2 for office BP (*p* < 0.001), 3.75 g/m2 for BP on HBPM (*p* < 0.001), and 2.96 g/m2 for awake BP on ABPM (*p* < 0.001). In a fully adjusted model that included all three BP measures, higher systolic BP measured by HBPM was associated with higher LVMI (3.94 g/m2, *p* < 0.001), whereas office systolic BP (−0.47 g/m2, *p* = 0.63) and awake systolic BP on ABPM (0.17 g/m2, *p* = 0.87) were not. At each of the 3 visits, office BP was also measured 3 times using a BpTRU BPM-200 oscillometric device and 3 times using the same Omron oscillometric devices used for HBPM. The results were similar when office BP was based on these devices. The results were also similar when using asleep or 24-hour systolic BP measured by ABPM and using diastolic BP instead of systolic BP. To consider the “true” values of office BP, BP on ABPM, and BP on HBPM, we also conducted additional analyses that estimated the associations among the different BP measures and their associations with target end-organ damage, correcting for regression dilution bias. The results were also similar. Figure 2 shows a summary of the results from the IDH study.

The results support a new paradigm in the US: when office BP, ABPM, and HBPM are conducted using guideline-recommended approaches, HBPM is superior to ABPM and office BP, and ABPM is not needed when office BP and HBPM are both performed. Therefore, HBPM may be a better reference standard for out-of-office BP monitoring than ABPM. These results are similar to those from a study by Jula et al., which compared the associations of office BP (4 visits with 2 readings/visit), BP measured by ABPM (every 15 min during the day and 30 min during the night over 24 h), and HBPM (2 AM readings and 2 PM readings per day for 7 days) with LVMI among 233 Finnish adults (35 to 54 years, mean age 46 years) with screening systolic/diastolic BP 180-220/100-120 mm Hg and not taking antihypertensive medication [50]. In a model with all 3 BP measures, only BP assessed by HBPM was associated with LVMI. This study did not compare the reliability of the BP measures. An argument in support of ABPM is that in contrast to HBPM, it measures asleep BP. Some studies have shown that asleep BP has a stronger association with outcomes than awake BP measured by ABPM [51–53]. In the IDH study, HBPM was superior to ABPM, even when using asleep BP or 24-hour BP. Table 2 provides a scientific rationale for why HBPM may be a better reference standard for out-of-office BP than ABPM. The central rationale for HBPM being superior to ABPM is that HBPM may provide a more reliable estimate of the resting BP outside of the office. HBPM is performed over a longer period of time under standardized conditions (i.e., resting and seated), which may average out the day-to-day variability in BP. In contrast, ABPM is performed over a shorter period of time (i.e., a 24-hour period) and is not typically done in a standardized manner and therefore may not be an ideal measure of resting BP. Additional studies in the US comparing HBPM to ABPM should be conducted that include individuals both not taking and taking antihypertensive medication, older adults, individuals with and without high office BP, and participants across several racial and ethnic groups.

**Table 2:** Rationale why HBPM may be a better reference standard than ABPM

Resting (or basal) BP outside of the office may be what is most strongly associated with target end-organ damage and CVD risk.
ABPM, performed over a single 24-hour period, is not done in a standardized manner and, except perhaps for sleep, does not measure resting BP.
HBPM is performed over several days (i.e. 7 days), while seated and resting at home and averages out the day-to-day variability in BP.
Therefore, compared to ABPM, HBPM may provide a more reliable estimate of resting BP outside of the office.

## Summary

In the US, although it is unclear whether ABPM is superior to HBPM or vice versa for predicting CVD, US guidelines and scientific statements have recommended ABPM as the preferred approach, particularly for adults not taking antihypertensive medication and for whom white coat hypertension or masked hypertension are being excluded. Because of practicality issues, the 2017 ACC/AHA BP guideline recommends that HBPM be the preferred initial approach, particularly for adults taking antihypertensive medication for whom white-coat effect and masked uncontrolled hypertension are being excluded.

More evidence demonstrating that HBPM is superior to ABPM would have a great impact on health care in the US since HBPM is a more feasible approach for both diagnosing and managing hypertension than ABPM. Despite the promise of HBPM, there are several barriers to its successful implementation in the US. Patient-level barriers include performing HBPM protocols over a long period of time, lack of education about the benefits of HBPM, lack of support from providers, and the financial costs of HBPM devices. Clinician-level barriers include concerns about device inaccuracy, concerns about low adherence to HBPM by patients, concerns about patient anxiety, increased burden on clinical staff and practices, additional time commitment to interpret BP readings, and lack of reimbursement for HBPM devices. Health care system-level barriers include a lack of integrated systems allowing BP readings to be transferred from HBPM devices to the electronic health record and a lack of a system for effectively administering cointerventions to patients.

In a 2020 Joint Policy Statement from the American Heart Association and American Medical Association, the authors endorsed important priorities to increase HBPM use in the US [24]. Over time, it is anticipated that HBPM will have a primary role in the routine diagnosis and management of hypertension in the US as more data supporting HBPM are acquired.
